# Prediagnostic Plasma Vitamin D Metabolites and Mortality among Patients with Prostate Cancer

**DOI:** 10.1371/journal.pone.0018625

**Published:** 2011-04-06

**Authors:** Fang Fang, Julie L. Kasperzyk, Irene Shui, Whitney Hendrickson, Bruce W. Hollis, Katja Fall, Jing Ma, J. Michael Gaziano, Meir J. Stampfer, Lorelei A. Mucci, Edward Giovannucci

**Affiliations:** 1 Channing Laboratory, Department of Medicine, Brigham and Women's Hospital and Harvard Medical School, Boston, Massachusetts, United States of America; 2 Department of Medical Epidemiology and Biostatistics, Karolinska Institutet, Stockholm, Sweden; 3 Department of Epidemiology, Harvard School of Public Health, Boston, Massachusetts, United States of America; 4 Department of Pediatrics, Medical University of South Carolina, Charleston, South Carolina, United States of America; 5 Clinical Epidemiology and Biostatistics Unit, Örebro University Hospital, Örebro, Sweden; 6 Divisions of Preventive Medicine and Aging, Department of Medicine, Brigham and Women's Hospital, Boston, Massachusetts, United States of America; 7 Massachusetts Veteran's Epidemiology, Research and Information Center, Geriatric Research Education and Clinical Center, VA Boston Healthcare System, Boston, Massachusetts, United States of America; 8 Department of Nutrition, Harvard School of Public Health, Boston, Massachusetts, United States of America; Genentech Inc., United States of America

## Abstract

**Background:**

Laboratory evidence suggests that vitamin D might influence prostate cancer prognosis.

**Methodology/Principal Findings:**

We examined the associations between prediagnostic plasma levels of 25(OH)vitamin D [25(OH)D] and 1,25(OH)_2_vitamin D [1,25(OH)_2_D] and mortality among 1822 participants of the Health Professionals Follow-up Study and Physicians' Health Study who were diagnosed with prostate cancer. Cox proportional hazards models were used to calculate hazard ratios (HRs) and 95% confidence intervals (CIs) of total mortality (n = 595) and lethal prostate cancer (death from prostate cancer or development of bone metastases; n = 202). In models adjusted for age at diagnosis, BMI, physical activity, and smoking, we observed a HR of 1.22 (95% CI: 0.97, 1.54) for total mortality, comparing men in the lowest to the highest quartile of 25(OH)D. There was no association between 1,25(OH)_2_D and total mortality. Men with the lowest 25(OH)D quartile were more likely to die of their cancer (HR: 1.59; 95% CI: 1.06, 2.39) compared to those in the highest quartile (P_trend_ = 0.006). This association was largely explained by the association between low 25(OH)D levels and advanced cancer stage and higher Gleason score, suggesting that these variables may mediate the influence of 25(OH)D on prognosis. The association also tended to be stronger among patients with samples collected within five years of cancer diagnosis. 1,25(OH)_2_D levels were not associated with lethal prostate cancer.

**Conclusions/Significance:**

Although potential bias of less advanced disease due to more screening activity among men with high 25(OH)D levels cannot be ruled out, higher prediagnostic plasma 25(OH)D might be associated with improved prostate cancer prognosis.

## Introduction

The first proposal of the hypothesis that vitamin D protects against prostate cancer appeared already two decades ago [1], followed by experimental studies suggesting that vitamin D may play an important role in the natural history of prostate cancer, not only for occurrence but also for disease progression through invasion, angiogenesis, and metastases [2]–[4]. Most epidemiological studies examining the associations between circulating levels of prediagnostic vitamin D metabolites (25(OH)vitamin D [25(OH)D] and 1,25(OH)_2_vitamin D [1,25(OH)_2_D]) and prostate cancer risk have reported null results [5]–[12]. Human studies have focused more recently on possible influences of vitamin D in prostate cancer progression, including a Norwegian study showing that a higher level of serum 25(OH)D after diagnosis was associated with a better prognosis of prostate cancer [13]. However, in that study blood samples were collected after prostate cancer diagnosis, and thus the 25(OH)D levels might already have been influenced by the cancer, by treatment, or by alterations in outdoor activities or diet after the cancer diagnosis. Further support for a possible influence of vitamin D on prognosis comes from studies showing that genetic variants in the vitamin D receptor are associated with Gleason score [14], and genetic variants in the vitamin D pathway are associated with risk of recurrence or progression and prostate cancer-specific mortality [15]. In the present study, we examined the hypothesis that higher plasma levels of prediagnostic vitamin D metabolites, 25(OH)D and 1,25(OH)_2_D, are associated with better prognosis of prostate cancer in two prospective cohort studies: the Health Professionals Follow-up Study and the Physicians' Health Study.

## Materials and Methods

### Ethics Statement

The Human Subjects Committee at the Harvard School of Public Health and the Human Subjects Committee at Brigham and Women's Hospital approved this study. Written consent was given by the patients for their information to be stored in the hospital database and used for research.

### Study populations

#### Health Professionals Follow-up Study (HPFS)

The HPFS is an ongoing prospective cohort study of 51,529 US men who were aged 40–75 years at enrollment in 1986, and followed through biennial questionnaires [16]. The participants reported information on height (at baseline only), body weight, physical activity and diet every two or four years. In 1993–1995, 18,018 men in the cohort donated a blood sample. Participants with a diagnosis of any cancer except non-melanoma skin cancer prior to the blood draw were excluded from the present analysis.

For each man who reported a prostate cancer diagnosis on a follow-up questionnaire or for whom a death certificate indicated that the cause of death was prostate cancer, we obtained medical and pathology records after receiving written permission from the participants or their next-of-kin. The response rate was 96% for nonfatal cases and we estimate having ascertained >98% of fatal cases [17]. Medical records and pathology reports were successfully obtained for 90% of the cases in the cohort. A study investigator blinded to questionnaire and assay information reviewed the medical records to confirm adenocarcinoma of the prostate and to abstract information on stage (according to the TNM staging scheme) and Gleason sum of the tumor. Patients without pathologic staging were classified as indeterminate stage unless there was clinical evidence of distant metastases at diagnosis.

#### Physicians' Health Study (PHS)

The PHS I was a randomized, double-blind, placebo-controlled trials of aspirin and β-carotene among 22,071 healthy US male physicians, aged 40–84 years, that began in 1982 [18]. Study participants provided baseline information via self-administrated questionnaires. Before randomization, 14,916 men (68%) provided a blood sample [7], and more than 70% of the specimens were received during September-November in 1982. Additional questionnaires were mailed at 6 months, 12 months, and annually thereafter to obtain medical information, including data on height, body weight and physical activity. Study investigators, blinded to the questionnaire or assay data, verified the reports of prostate cancer by participants and reviewed medical records and pathology reports to determine the tumor Gleason score and stage (according to the TNM classification scheme). If pathologic staging was not available, cases were classified as indeterminate stage unless there was clinical evidence of distant metastases at the time of diagnosis.

### Laboratory measurements

#### Vitamin D Metabolites

Plasma concentrations of 1,25(OH)_2_D and 25(OH)D were determined by radioimmunosorbant assay in the laboratory of Dr. Bruce Hollis [19], [20]. In the HPFS, cases were assayed in four separate analytical runs (cases up to January 1996, February 1996-January 1998, February 1998-January 2000, and February 2000–January 2004) [21], [22]. The PHS cases were assayed in two analytic runs [23], [24]. The mean intrapair coefficients of variation from blinded quality control samples ranged from 5–10% for both 25(OH)D and 1,25(OH)_2_D in PHS and HPFS [21]–[24].

To assess the intraperson consistency of vitamin D metabolite concentrations over time, we measured 1,25(OH)_2_D and 25(OH)D for 144 men in the HPFS who were free of cancer diagnosis and who provided a blood sample in 1993/1994 and again in 1997 (mean of 3.03±0.46 years apart) [21]. Adjusting for age, race, and season of the year, the Pearson correlation coefficients between the two time points were 0.50 (P<0.0001) for 1,25(OH)_2_D and 0.70 (P<0.0001) for 25(OH)D.

### Follow-up of participants with prostate cancer

From the two cohorts, we identified a total of 1822 men diagnosed with prostate cancer after blood collection on whom circulating levels of vitamin D had been previously measured as part of nested case-control studies of vitamin D metabolites and risk of incident prostate cancer; 1318 in HPFS (diagnosed during 1993–2004) and 504 in PHS (diagnosed during 1982–1998) [21]–[24]. A total of 185 patients (10.2%) were diagnosed within 2 years after blood collection. All cancer patients were followed from the date of cancer diagnosis until date of death, bone metastasis, or end of the current follow-up (July 17, 2009 in HPFS and March 31, 2009 in PHS), whichever came first. Information on the course of prostate cancer was collected through follow-up questionnaires, which collected treatments and development of metastases. When a metastasis was reported, we contacted the treating physician of the patient and requested a review of the relevant medical records to verify the diagnosis. Consent was obtained from the participants to review the medical records. Information on date and cause of death was obtained from repeated mailings and searches on the National Death Index and confirmed through review of death certificates and medical records. Deaths due to prostate cancer were based on evidence of metastases and disease progression, with no other plausible cause of death. A bone metastasis or death from prostate cancer was taken as the end point when calculating hazard ratios for prostate cancer-specific mortality; death from any cause was used as the endpoint when calculating hazard ratios for total mortality.

### Statistical analysis

Because vitamin D metabolites were assayed in different batches from blood samples collected at various seasons, and the vitamin D metabolites levels (especially 25(OH)D) vary largely with season, we calculated the batch-, season-, and cohort-specific quartiles for 1,25(OH)_2_D and 25(OH)D. Seasons were classified as follows: summer (June-August), autumn (September–November), spring (March–May), and winter (December–February). In HPFS, the mean (standard deviation) values of the quartiles standardized for batch and season as described by Rosner et al. [25] are 20.36 (6.83), 30.24 (3.72), 37.26 (4.88) and 49.03 (10.04) pg/ml for 1,25(OH)_2_D; and 16.19 (4.46), 23.51 (2.24), 28.78 (3.16), and 38.35 (7.18) ng/ml for 25(OH)D. Similarly, in PHS, 24.10 (3.23), 29.84 (1.40), 34.66 (1.60), and 44.47 (6.59) pg/ml for 1,25(OH)_2_D; and 17.55 (3.98), 25.42 (2.38), 31.12 (2.39), and 42.78 (7.84) ng/ml for 25(OH)D. We used ANOVA tests to examine the differences in the means of age at blood draw, age at cancer diagnosis, and follow-up time across the quartiles of vitamin D metabolites. A two-way chi-square test was used to obtain *P* values for the associations of Gleason score, TNM stage, smoking status, body mass index (BMI), and physical activity with the quartiles of vitamin D metabolites.

Cox proportional hazards regression models were used to estimate hazard ratios (HRs) and their 95% confidence intervals (CIs) to assess the relation of plasma vitamin D metabolites with total and prostate cancer-specific mortality. The highest quartile was used as the reference group for both 1,25(OH)_2_D and 25(OH)D. We tested for trends across the quartiles of 25(OH)D and 1,25(OH)_2_D treating the quartiles (1,2,3,4) as a continuous variable. We hypothesized that tumor grade and stage might be the intermediate factors relating vitamin D to prostate cancer progression; therefore we adjusted first for age at diagnosis, smoking (never vs. ever), BMI (<25, ≥25 and <28, or ≥28 kg/m^2^), and physical activity (low, moderate, or high) obtained from the questionnaire collected before the blood donation. In additional models, we further adjusted for Gleason score (<7, 7, >7, or missing) and TNM stage (T1/T2, T3, T4/N1/M1, or missing) to assess the potential of vitamin D metabolites in predicting cancer prognosis beyond these tumor characteristics. The proportional hazard assumption was checked and found to hold for all covariates. Analyses were first conducted among these two cohorts separately. Because the findings were similar, we combined these two cohorts to increase statistical power and we report only results obtained from the pooled analyses.

We further stratified the analyses by age at diagnosis (<65 or ≥65 years), tumor stage (TNM: T1/T2 or T3/T4/N1/M1), and Gleason score (<7 or ≥7). To alleviate concern of reverse causality (i.e., prostate cancer influencing the levels of vitamin D metabolites), in a sub-analysis, we excluded patients with samples collected <2 years before prostate cancer diagnosis. We also conducted stratified analyses by the time interval between blood draw and prostate cancer diagnosis (<5 or ≥5 years). All P values are two-sided and considered statistically significant at P < 0.05. All analyses were performed using SAS, version 9.1 (SAS Institute, Cary, NC, USA).

## Results

The mean age (standard deviation, SD) at blood draw of participants in the analysis was 63.7 (7.8) years in HPFS and 59.2 (7.6) years in PHS. The mean age (SD) at cancer diagnosis was 69.5 (7.4) years in HPFS and 67.8 (6.5) years in PHS. Characteristics of the prostate cancer patients in the combined analyses are presented in **Table 1**.

**Table 1:** Baseline Characteristics of 1822 men with prostate cancer according to Quartiles of Plasma 25(OH)D and 1,25(OH)_2_D, HPFS (1993) and PHS (1982).

	25(OH)D					1,25(OH)_2_D				
	Q1 (low)	Q2	Q3	Q4 (high)	p value	Q1 (low)	Q2	Q3	Q4 (high)	p value
**No. of cases**	468	458	450	446		470	449	458	445	
**Age at blood draw, yrs**	62.8 (8.1)	62.2 (8.1)	62.6 (7.6)	62.2 (8.2)	0.42^1^	63.5 (8.2)	62.6 (7.7)	61.8 (7.9)	61.9 (8.1)	0.0007^1^
**Age at diagnosis, yrs**	69.2 (7.2)	68.8 (7.3)	69.2 (7.1)	68.9 (7.3)	0.77^1^	70.0 (7.3)	69.3 (6.9)	68.4 (7.3)	68.3 (7.4)	<0.0001^1^
**Mean follow-up, yrs**	9.7 (4.3)	9.9 (4.4)	10.2 (4.1)	10.0 (4.5)	0.13^1^	9.7 (4.2)	10.0 (4.4)	10.0 (4.3)	10.0 (4.5)	0.33^1^
**Gleason score**										
Gleason <7	222 (47.4)	200 (43.7)	252 (56.0)	233 (52.2)		237 (50.4)	215 (47.9)	221 (48.3)	234 (52.6)	
Gleason = 7	136 (29.1)	155 (33.8)	130 (28.9)	129 (28.9)		148 (31.5)	135 (30.1)	142 (31.0)	125 (28.1)	
Gleason >7	61 (13.0)	50 (10.9)	36 (8.0)	47 (10.5)	0.02^2^	48 (10.2)	50 (11.1)	50 (10.9)	46 (10.3)	0.89^2^
Missing	49 (10.5)	53 (11.6)	32 (7.1)	37 (8.3)		37 (7.9)	49 (10.9)	45 (9.8)	40 (9.0)	
**TNM stage**										
T1/T2	335 (71.6)	326 (71.2)	348 (77.3)	346 (77.6)		332 (70.6)	338 (75.3)	346 (75.6)	339 (76.2)	
T3	57 (12.2)	61 (13.3)	53 (11.8)	46 (10.3)		67 (14.3)	51 (11.4)	51 (11.1)	48 (10.8)	
T4/N1/M1	26 (5.6)	22 (4.8)	14 (3.1)	16 (3.6)	0.26^2^	23 (4.9)	14 (3.1)	23 (5.0)	18 (4.0)	0.36^2^
Missing	50 (10.7)	49 (10.7)	35 (7.8)	38 (8.5)		48 (10.2)	46 (10.2)	38 (8.3)	40 (9.0)	
**Smoking status**										
Never	225 (48.1)	245 (53.5)	208 (46.2)	215 (48.2)		239 (50.8)	215 (47.9)	229 (50.0)	210 (47.2)	
Ever	243 (51.9)	213 (46.5)	242 (53.8)	231 (51.8)	0.15^2^	231 (49.2)	234 (52.1)	229 (50.0)	235 (52.8)	0.65^2^
**Body mass index (kg/m** 2 **)**										
<25	200 (42.7)	216 (47.2)	211 (46.9)	244 (54.7)		205 (43.6)	208 (46.3)	223 (48.7)	235 (52.8)	
≥25 and <28	152 (32.5)	154 (33.6)	163 (36.2)	152 (34.1)		159 (33.8)	154 (34.3)	163 (35.6)	145 (32.6)	
≥28	116 (24.8)	88 (19.2)	76 (16.9)	50 (11.2)	<0.0001^2^	106 (22.6)	87 (19.4)	72 (15.7)	65 (14.6)	0.02^2^
**Physical activity**										
Low	212 (45.3)	161 (35.2)	159 (35.3)	132 (29.6)		188 (40.0)	151 (33.6)	162 (35.4)	163 (36.6)	
Moderate	151 (32.3)	157 (34.3)	151 (33.6)	143 (32.1)		158 (33.6)	151 (33.6)	162 (35.4)	131 (29.4)	
High	104 (22.2)	139 (30.4)	138 (30.7)	169 (37.9)	<0.0001^2^	122 (26.0)	146 (32.5)	132 (28.8)	150 (33.7)	0.09^2^
Missing	1 (0.2)	1 (0.2)	2 (0.4)	2 (0.4)		2 (0.4)	1 (0.2)	2 (0.4)	1 (0.2)	
**No. of deaths**	171	147	143	134	0.19^2^	168	143	139	145	0.35^2^
**No. of lethal cases**	64	57	41	40	0.05^2^	55	47	52	48	0.93^2^

1 ANOVA test.

2 Chi-square test. Individuals with missing values were excluded from test.

Higher 25(OH)D levels were associated with lower BMI (P<0.0001), more physical activity (P<0.0001), and also lower Gleason score at prostate cancer diagnosis (P = 0.02) (**Table 1**). Although not statistically significant, higher 25(OH)D levels were associated with a lower frequency of metastatic prostate cancer. Higher 1,25(OH)_2_D level was associated with younger age (P = 0.0007) as well as lower BMI (P = 0.02). During follow-up, we documented 595 deaths including 166 cases of death from prostate cancer, and 36 men living with bone metastasis; we combined the prostate cancer deaths and metastatic cases into a single category of lethal prostate cancer (n = 202).

Among the men with prostate cancer, those with the lowest levels of 25(OH)D based on quartiles had a slightly increased total mortality compared to patients with the highest quartile. After adjustment for age at diagnosis, BMI, physical activity, and smoking, the HR was 1.22 (95% CI: 0.97, 1.54); a test for linear trend was suggestive, but not statistically significant (P_trend_ = 0.07) (**Table 2**).

**Table 2:** Hazards ratios (HRs) and 95% confidence intervals (CIs) for total mortality and lethal prostate cancer among prostate cancer patients by quartiles of vitamin D metabolites, combining HPFS and PHS (N = 1822).

	Total mortality				
	No.	Rate per 1000 person-years^1^	HR1^2^ (95% CI)	HR2^2^ (95% CI)	HR3^2^ (95% CI)
**25(OH)D**					
Q1 (low)	171	37.9	1.22 (0.97–1.54)	1.16 (0.92–1.47)	1.10 (0.87–1.39)
Q2	147	32.4	1.09 (0.86–1.38)	1.06 (0.83–1.34)	1.05 (0.83–1.34)
Q3	143	31.2	1.01 (0.79–1.28)	1.06 (0.84–1.35)	1.06 (0.83–1.34)
Q4 (high)	134	30.0	1.00	1.00	1.00
P_trend_			0.07	0.23	0.46
**1,25(OH)_2_D**					
Q1 (low)	168	36.7	0.97 (0.77–1.22)	0.96 (0.77–1.21)	0.92 (0.73–1.16)
Q2	143	32.0	0.89 (0.70–1.13)	0.88 (0.69–1.11)	0.88 (0.69–1.11)
Q3	139	30.3	0.94 (0.74–1.18)	0.92 (0.73–1.16)	0.93 (0.73–1.17)
Q4 (high)	145	32.5	1.00	1.00	1.00
P_trend_			0.72	0.70	0.43

	Lethal Prostate cancer				
	No.	Rate per 1000 person-years^1^	HR1^2^ (95% CI)	HR2^2^ (95% CI)	HR3^2^ (95% CI)
**25(OH)D**					
Q1 (low)	64	14.2	**1.59 (1.06-2.39)**	1.46 (0.97–2.20)	1.31 (0.86–1.99)
Q2	57	12.6	1.43 (0.95–2.16)	1.31 (0.87–1.98)	1.32 (0.87–2.00)
Q3	41	9.0	0.98 (0.63–1.52)	1.14 (0.74–1.78)	1.09 (0.70–1.70)
Q4 (high)	40	9.0	1.00	1.00	1.00
P_trend_			**0.006**	0.06	0.14
**1,25(OH)_2_D**					
Q1 (low)	55	12.0	0.95 (0.64–1.41)	0.95 (0.63–1.41)	0.85 (0.57–1.27)
Q2	47	10.5	0.87 (0.58–1.31)	0.84 (0.56–1.26)	0.89 (0.59–1.34)
Q3	52	11.3	1.01 (0.68–1.49)	0.95 (0.64–1.41)	0.97 (0.65–1.45)
Q4 (high)	48	10.8	1.00	1.00	1.00
P_trend_			0.66	0.68	0.38

1 Crude mortality rate.

2 HR1: adjusted for age at diagnosis, body mass index, physical activity, and smoking; HR2: adjusted for age at diagnosis, body mass index, physical activity, smoking, and Gleason score; HR3: adjusted for age at diagnosis, body mass index, physical activity, smoking, Gleason score, and TNM stage; 25(OH)D and 1,25(OH)_2_D were mutually adjusted.

Men in the lowest quartile of 25(OH)D had a higher risk of lethal prostate cancer, after adjustment for age at diagnosis, BMI, physical activity, and smoking (HR: 1.59; 95% CI: 1.06, 2.39); a statistically significant dose-response relationship was also observed (P = 0.006) (**Table 2**). This association was largely explained by the association between 25(OH)D levels and advanced stage and higher Gleason score; after adjustment for those factors, the association was diminished and no longer significant (**Table 2**). No association was observed for 1,25(OH)_2_D with either total mortality or lethal prostate cancer (**Table 2**). In separate analyses by cohort, after adjustment for age at diagnosis, physical activity, and smoking, the lowest quartile of 25(OH)D was statistically significantly associated with a higher risk of lethal prostate cancer in HPFS (HR: 2.01; 95% CI: 1.12, 3.59), but the association was not as apparent in PHS (HR: 1.17; 95% CI: 0.66, 2.09).

In analyses excluding patients with prostate cancer diagnosed within 2 years after blood draw, we observed a diminished association between 25(OH)D and lethal prostate cancer, after adjustment for age at diagnosis, BMI, physical activity, and smoking (HR: 1.42; 95% CI: 0.91, 2.22). Stratified analyses by the time interval between blood draw and prostate cancer diagnosis showed an association between 25(OH)D and lethal prostate cancer only among men with blood sample collected <5 years before cancer diagnosis (HR: 1.90; 95% CI: 1.07, 3.38), but not among those with blood sample collected ≥5 years before cancer diagnosis (HR: 1.22; 95% CI: 0.66, 2.27).We found no material differences in the associations stratified by age, stage at diagnosis, or Gleason score (data not shown).

## Discussion

Using prospectively collected plasma samples, we found that prostate cancer patients with a lower concentration of prediagnostic plasma 25(OH)D had a higher risk of developing metastatic or fatal (lethal) prostate cancer. Circulating 1,25(OH)_2_D level was not associated with either total mortality or lethal prostate cancer. The association between 25(OH)D and prostate cancer-specific mortality appeared to be mediated mainly through Gleason score and TNM stage at diagnosis; after adjustment for these tumor characteristics, the associations were no longer statistically significant. Such mediation is biologically plausible since vitamin D promotes differentiation and apoptosis while inhibiting angiogenesis and proliferation [2]–[4], [26], processes that can affect tumor grade and stage. Accordingly, statistical adjustment for Gleason score and TNM stage would tend to lead to null results. Our results suggest that 25(OH)D level may be biologically and clinically relevant, but this level may not add prognostic value in addition to tumor stage and grade. The different results between 25(OH)D and 1,25(OH)_2_D might be due to the fact that levels of 1,25(OH)_2_D are known to be tightly regulated and circulating 1,25(OH)_2_D does not reflect nutritional status of vitamin D except at severe deficiency states and hence probably does not assess of intracellular 1,25(OH)_2_D in the prostate.

The inverse association between 25(OH)D level and lethal prostate cancer was only seen among men with blood samples collected less than five years before cancer diagnosis. This finding may reflect that 25(OH)D level at late stages of tumor development modifies prostate cancer prognosis. This stronger association among men with more recent blood draw could potentially explain the stronger association observed in the HPFS compared to the PHS, since there was on average a shorter time interval between blood draw and prostate cancer diagnosis, i.e., more of the prostate cancers in HPFS were diagnosed within five years after the blood draw. On the other hand, this finding might also reflect a possibility of reverse causality, i.e., a preclinical occult cancer might reduce 25(OH)D levels. However, most cases were diagnosed at an early stage, and results persisted when the analyses were limited to cancers diagnosed at early stage (among patients with either Gleason score <7 or TNM stage in T1/T2, a HR of 1.64 [95% CI: 0.90, 2.98] was observed). If reverse causality entirely explained our findings we might have expected that prediagnostic 25(OH)D was more strongly predictive of outcome for advanced stage disease than localized disease at diagnosis, but we did not observed this. Further, although somewhat attenuated, associations were still observed after excluding those who had blood samples collected within two years of diagnosis. We believe that our medical records review for metastasis cases was rather comprehensive, while it is still possible that we misclassified or missed some metastases (e.g., men died before questionnaire was sent). Such misclassification or missingness should however be largely non-differential with respect to prediagnostic vitamin D levels and theoretically only lead to an underestimation of the real association.

We considered whether the association between 25(OH)D and lethal prostate cancer may have been explained by confounding or bias. Although confounding cannot be ruled out, we controlled for BMI and physical activity, which influence vitamin D levels and may affect prostate cancer prognosis. Also possible is that participants with higher 25(OH)D levels may have more frequent prostate cancer screening compared to those with lower levels, leading to the detection of more indolent, earlier staged tumors. Participants with higher 25(OH)D levels are presumably more health conscious compared with participants with lower levels, this is evidenced in our data as high 25(OH)D level was associated with more outdoor physical activities. Health consciousness is related to the participation in prostate cancer screening; for example, according to a national survey, physician counsel and patient prevention consciousness are the major influences when a patient decides to attend prostate carcinoma testing in the United States [27]. However, the rate of PSA screening tends to be uniformly high in these cohorts of physicians and health professionals. For example, approximately 80% of the prostate cancer patients in the HPFS had at least one PSA screening test before blood donation [7]. In addition, participation in PSA screening did not seem to be associated with 25(OH)D levels in our data. For instance, PSA screening did not differ across quartiles of 25(OH)D in HPFS (Chi-square test; P = 0.41).

Although we had only one measurement for the vitamin D metabolites, the good correlation between two measures of both metabolites taken three years apart among 144 men suggests that one measurement of the circulating levels is reasonably representative. Another limitation of the present study is that the plasma samples were collected in different seasons, and samples from the two cohorts were measured at six separate times, years apart, albeit in the same laboratory. We used the season-, batch-, and cohort-specific cutoffs to define the quartiles of vitamin D metabolites, preventing the possibility of comparing absolute levels of the metabolites with other studies. Despite the batch and seasonal variations in absolute levels of 25(OH)D, each of the within-batch coefficients of variation ranged from 5 to 10%, and thus ranking by 25(OH)D status was acceptable.

Other data on vitamin D and prostate cancer prognosis are sparse. A previous study from Norway observed a strong association between low level of serum 25(OH)D and a higher risk of death from prostate cancer [13]. Using serum samples collected either at the time of or after cancer diagnosis could potentially be problematic. As the authors discussed, the disease status might have influenced 25(OH)D concentration due to reduced outdoor physical activity and less vitamin D supplementation [13]. The stronger association between 25(OH)D and better survival for prostate cancer among patients who had hormonal treatment before sample collection also supports this suspicion. In addition, treatment for prostate cancer could alter the vitamin D level as well as influencing cancer prognosis. Our study used samples collected before diagnosis, and thus avoids these limitations. Other support of the vitamin D pathway on prostate cancer prognosis is that genetic variants in the vitamin D receptor are associated with Gleason score [14], genetic variants in the vitamin D pathway are associated with risk of recurrence/progression and prostate cancer-specific mortality [15], and the high expression of the vitamin D receptor in prostatectomy specimens is associated with better prognosis [28]. Some but not all studies also have reported better prognosis of prostate cancer for cases diagnosed and presumably treated in the summer/autumn months, when 25(OH)D levels tend to be higher [29]–[31].

In summary, we observed an inverse association of 25(OH)D with risk of lethal prostate cancer. These data further support the potential influence of the vitamin D pathway on prostate cancer prognosis.
